# Heterologous Wharton's Jelly Derived Mesenchymal Stem Cells Application on a Large Chronic Skin Wound in a 6-Month-Old Filly

**DOI:** 10.3389/fvets.2019.00009

**Published:** 2019-01-30

**Authors:** Aliai Lanci, Barbara Merlo, Jole Mariella, Carolina Castagnetti, Eleonora Iacono

**Affiliations:** Department of Veterinary Medical Sciences, University of Bologna, Bologna, Italy

**Keywords:** mesenchymal stromal cells, Wharton's jelly, foal, wound, carboxymethylcellulose, plasma

## Abstract

A complex feedback of growth factors, secreted by a variety of cell types, is responsible for the mediation of skin healing. Despite the recent advances in wound healing management, this fails up to 50% and skin wounds can still be considered one of the main causes of morbidity, both in human and veterinary medicine. Regenerative medicine, involving mesenchymal stromal cells (MSCs), is nowadays a promising solution for skin wound healing. Indeed, MSCs are involved in the modulation of the inflammatory local response and cell replacing, by a paracrine mode of action. Local application of equine umbilical cord Wharton's jelly MSCs (WJMSCS) was carried out in a 6-months-old filly with a non-healing skin wound. Heterologous WJMSCs were applied four times using a carboxymethylcellulose (CMC) gel, produced dissolving CMC in autologous plasma. At first application the mean wound area was 7.28 ± 0.2 cm^2^. Four days after the last application of WJMSCs, the mean wound area was 1.90 ± 0.03 cm^2^, and the wound regression rate was +74%. No local or systemic side effects were registered after WJMSCs application and no evident exuberant scar was observed after wound healing. At discharge, the mean wound area was 0.38 ± 0.01 cm^2^ and the total regression rate was +80%. Five days later, the wound was completely healed. In the present clinical case report, the use of WJMSCs led to promising clinical results, paving the way for possible future applications in the treatment of chronic wounds in horses.

## Introduction

Persistent or slow wound healing are a challenge for clinicians, both in equine and human medicine. Frequently wound healing results in inadequate tissue reorganization and in a high re-injury rate (1), because of a breakable and susceptible to re-injury new epithelium (2). This could result in a long period of incapacity or in an unsatisfactory return to performance (1). Due to their properties, mesenchymal stem cells (MSCs) offer a great chance for cell-based therapies and tissue engineering applications. In equine medicine, the common sources of autologous and heterologous MSCs for clinical applications are bone marrow (BM) and adipose tissue (AT) (3). However, an invasive procedure is required and a large variability in the cell yield related to the donor was demonstrated for both these sources (4). Furthermore, adult MSCs have limited potential in terms of *in vitro* proliferation capability (5, 6) and do not appear to noticeably improve long-term functionality compared to MSCs derived from fetal adnexa (7). Fetal adnexa tissues and fluids can be considered as an important source of cells for regenerative medicine. They are easily procured, without invasive procedures for donors, and derived MSCs have intermediate characteristics between embryonic and adult stem cells. Indeed, MSCs derived from fetal adnexa seem to preserve some characteristics typical of the primitive native layers (8); on the other hand, these cells have not shown tumorigenicity, thus making them an exciting source of regenerative cells (9). Among fetal adnexal tissues, the major sources of MSCs are amniotic membrane and Wharton's jelly (WJ) (10). Usually, clinical treatments based on MSCs use provide for their parenteral injection; however only a small amount of the injected MSCs engraft successfully (11). In veterinary medicine, in the last few years, good results in the skin healing were obtained using local application of adipose derived MSCs (12), local injection of peripheral blood (13, 14), and local application of amniotic fluid derived MSCs (15, 16). As recently demonstrated by our research group, equine WJ could be considered as a viable source of MSCs, with reliable migration capacities. Furthermore, due to WJMSCs molecular characteristics, such as the lack of expression of MCH-I and MCH-II, WJ can be considered as a convenient cell source for autologous or allogeneic applications. Here we described a clinical case of a 6-months-old filly, hospitalized for a re-injury of a pressure wound treated using local application of heterologous WJMSCs.

## Case Details

### Signalment, History, and Clinical Findings

A Standardbred filly, born at the Equine Perinatology and Reproduction Unit (UPER), Department of Veterinary Medical Sciences (DIMEVET), University of Bologna, at birth presented severe hind limbs angular and extensor deformities and she was unable to stand without help. Since she spent long periods in decubitus, 3 days after birth, a superficial pressure sore developed on the left hock. The wound was classified, as previously reported in foal (16), as a grade 4 by Sessing classification (17) and grade 3 by National Pressure Ulcer Advisory Panel (NPUAP) (18). This area was daily cleaned with sterile saline solution, treated with ozone ointment (RHD®, Acme Srl, Italy) and bandaged. At discharge (10 days after birth), the wound was classified as grade 3 by Sessing classification and grade 2 by NPUAP classification. At farm, the wound was treated with ozone ointment and it was completely healed at 2 months of foal's life. The owner and the farm's veterinarian referred that the newly formed skin appeared very thin with a subcutaneous serum collection. The filly continued to spend a long time in decubitus and a month later the wound reopened. The wound was then newly treated with ozone ointment, but without results; therefore, the filly was referred to UPER. At admission, the wound was classified as grade 5 by Sessing classification and 4 by NPUAP classification. It was photographed using a digital camera and its area (cm^2^) was obtained using ImageJ (Version 1.6). The area was measured three times and the average was calculated to obtain a single measure. The mean wound area at admission (T0) was 4.73 ± 0.15 cm^2^. The skin and subcutaneous tissue appeared necrotic.

### Treatment and Outcome

Since necrotic tissue prolongs inflammation and increases the risk of bacterial contamination, a deep surgical curettage was carried out. In equine species, this is the most effective method to improve wound healing (19). After surgical treatment, the wound was medicated with Dermaflon^TM^ (Zoetis S.r.l, Italy), to stimulate tissue regeneration, and bandaged. Eight days after removing necrotic tissue (T8), the mean wound area was 7.28 ± 0.20 cm^2^. The mean percentage of regression was calculated using the followed formula:

$$\begin{array}{l} \%\;\text{of regression}\;=\;\left(\text{A}\;-\;\text{a}\;x\;100\right)/\text{A} \end{array}$$

where *A* is the initial area and *a* is the same area measured at the end of the phase of treatment. After surgical curettage, the mean wound regression resulted negative (−54%) and the wound area was higher than at admission. Since the results obtained by our group applying MSCs for treating skin wound in neonatal foal (15, 16) and our recent findings on equine WJMSCs (20), we decided to treat the wound with heterologous WJMSCs. The written consent was given by the owner.

MSCs used in the present study were isolated from a WJ sample recovered at delivery from a mare of the same owner, housed at UPER for foal birth assistance. The mare was negative for equine infectious anemia (Coggins Test) and vaccinated for Tetanus, Equine Herpervirus and Influenza. Furthermore, at admission, a complete physical examination, transabdominal and transrectal ultrasound examination, complete blood count and biochemistry profile were carried out. Experimental procedures were approved by the Ethics Committee, University of Bologna and by the Ministry of Health (8134-X/10); a written consent was given by the owner to allow tissues recovery.

**Figure 1:**
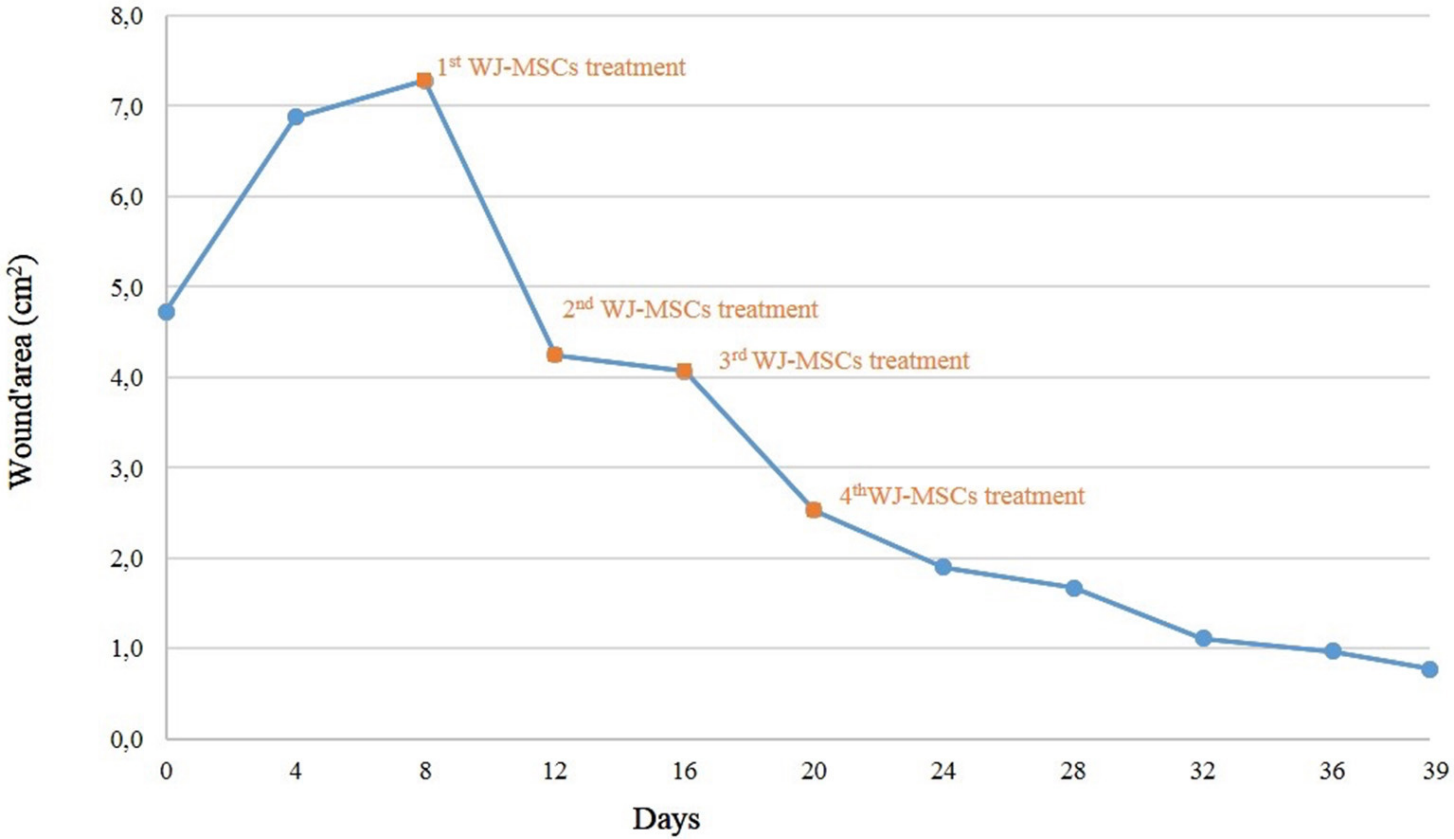
Trend of wound regression.

**Figure 2:**
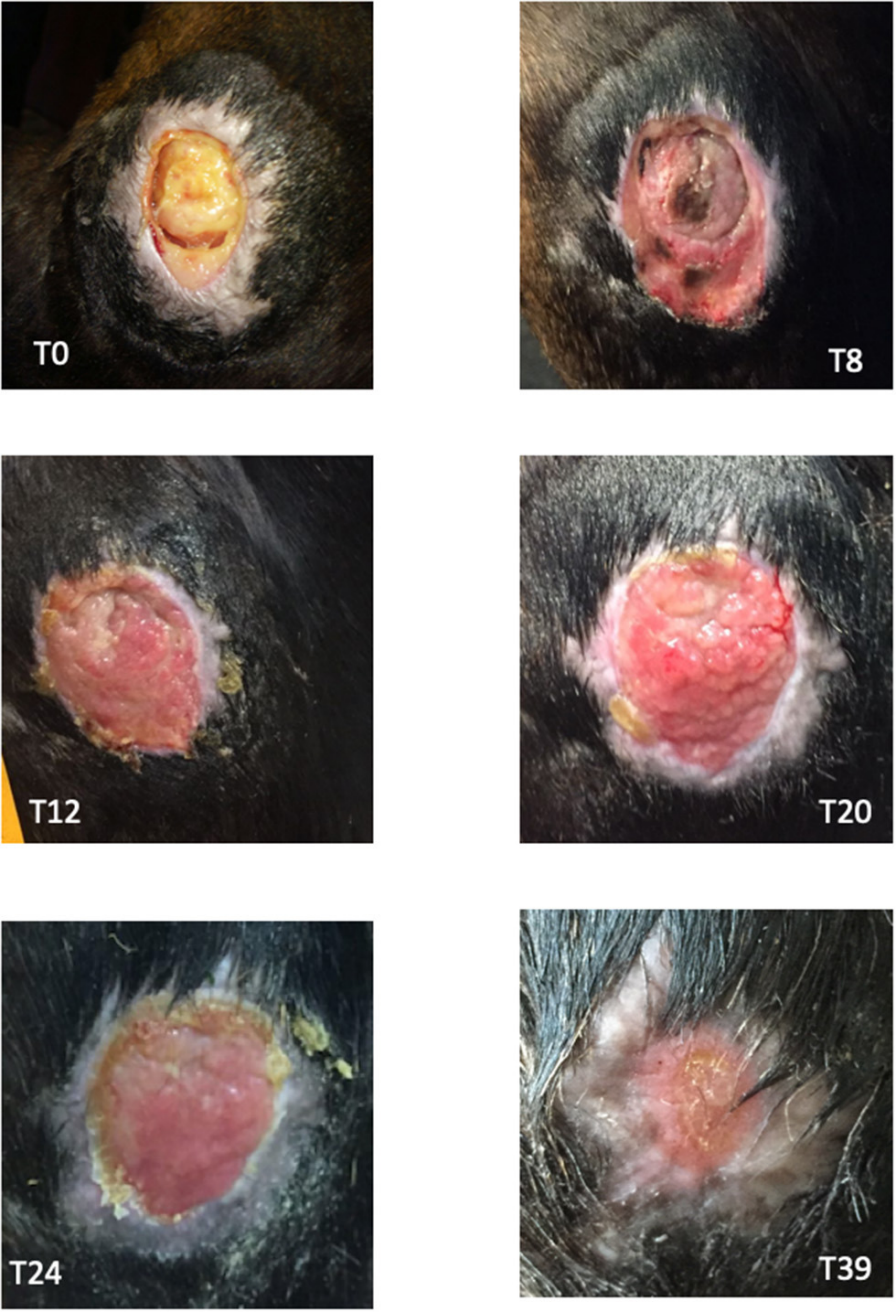
Images of the wound at different time points. T0: day of admission, before surgical curettage. T8: 8 days after the surgical curettage and before WJMSCs application. T12: 4 days after the first WJMSCs application (T8); granulation tissue was visible and no signs of rejection or inflammation were present. T20: after the third WJMSCs application. T24: after the last WJMSCs application. T39: day of discharge.

At the Laboratory of Reproduction and Animal Biotechnology, DIMEVET, University of Bologna, MSCs from WJ sample were isolated as previously described (20, 21). Briefly, under a laminal flow hood, umbilical cord was disinfected by immersing for few seconds in 70% ethanol and rinsed by repeated immersion in D-PBS. WJ was then isolated, weighed, minced finely by sterile scissors and fragments were incubated in a 37°C water bath for 1–2 h into a 50 mL polypropylene tube (Falcon^TM^), containing 1 mL/1 g sample of digestion solution [0.1% (w/v) collagenase type IV (Gibco, Invitrogen Corporation), in D-PBS]. The mixture was then filtered and collagenase was inactivated by diluting 1:1 in D-PBS, supplemented with 10% (v/v) FBS (Gibco). Nucleated cells were pelleted at 470 g for 10 min. After isolation, primary cells were plated in a 25 cm^2^ flask in 5 mL of culture medium [D-MEM-F12 Glutamax (Gibco), plus 10% v/v FBS, 100 IU/mL penicillin and 100 μg/mL streptomycin (Sigma Aldrich)]. Cells were incubated in a 5% CO_2_ humidified atmosphere at 38.5°C. At ~80–90% of confluence, they were dissociated by 0.25% trypsin, counted and plated at the concentration of 5 × 10^3^ cells/cm^2^ as “Passage 1” (P1), and so on for the subsequent passages. In the present study, cells at P3 of *in vitro* culture were used for clinical applications. *In vitro* differentiation and molecular characterization by PCR were carried out to confirm cell stemness (19, 20). Used cells resulted positive for CD90 and CD44 and were able to differentiate in adipogenic, chondrogenic, and osteogenic lineages as stated by Dominici et al. (22) and requested by Italian guide lines for using MSCs in veterinary medicine (GU Serie Generale n.277, 2013). Furthermore, before applications, mycoplasma contamination was excluded (GU Serie Generale n.277, 2013). For performing local application, a gel of carboxymethylcellulose (CMC) was produced as previously reported (16) using 5 mL of autologous plasma, collected with sterile closed system. Autologous plasma was used both as diluent and metabolic support. Immediately before applications, cells were added to the gel in sterile conditions, under a laminar flow hood. For each application, approximately 5 × 10^6^ WJMSCs were used. In surgery room, CMC + plasma gel was transferred over a sterile gauze moistened with isotonic sterile saline solution (0.9% NaCl). Before applying MSCs, the wound was rinsed with 0.9% NaCl sterile solution, and the skin around the wound was greased with Vaseline for preventing damages at the next dressing. Immediately after gel application, the limb was bandaged with cotton, gauze, and VetRap (3M, Milan, Italy). WJMSCs, in CMC + plasma gel, were applied every 4 days for 4 consecutive times (T8-T12-T16-T20). Before every MSCs application, the wound was photographed and the area was determined as stated in paragraph 2.1. Four days after the last application, no further bandages were applied and the wound was daily cleaned and treated with hydrotherapy using cold tap water for 10 min/day. Thirty-nine days after admission, the filly was discharged, and at home she was kept outside. The owner was recommended to continue hydrotherapy until the full wound healing. The trend of wound regression is plotted in Figure 1. A granulation tissue was visible after the first MSCs application, without signs of rejection or inflammation (Figure 2). The wound regression/time ratio reduced gradually, with a fast reduction between the first and second MSCs+CMC plasma gel applications (Figure 1). Four days after the last application of WJMSCs (T24), at the time of dressing, the mean wound area was 1.90 ± 0.03 cm^2^; the wound regression rate between T8 (first application) and T24 was +74%. At discharge (T39), the mean wound area was 0.38 ± 0.01 cm^2^ and the total regression rate, between T8 and T39, was +80%. Five days later, the wound was completely healed, as referred by the owner. No evident exuberant scar was present after wound healing and the hair grew completely without changing color (Figure 2; Figure S1). After discharge, the owner did not refer other relapses. No skin biopsy was carried out, given the concern of the owner about a possible relapse.

## Discussion

Wound healing below the hock or knee could be a problem even in the healthiest adult horses and foals (23). These wounds could be costly, time-consuming to manage and may lead to decreased performance, withdrawal from the races or euthanasia (24). This condition is further amplified in foals, because of their thin skin and prolonged decubitus (25).

In this study, a CMC+ plasma gel was tested as scaffold for local application of equine WJMSCs in a 6-months-old filly, referred to our hospital for a non-healing large skin wound. Due to their anti-inflammatory and immunomodulating properties, MSCs are important in the regenerative medicine field (13, 15, 16, 26). At initial investigation stages, studies were performed with BMMSCs; nowadays, it is known that WJMSCs may secrete many growth factors by a paracrine mechanism (27, 28). WJMSCs seem to possess more pro-angiogenic properties than BMMSCs (29) and higher IL-8 expression (30). IL-8 is involved in skin regeneration, since by directly influencing the endothelial cells, IL-8 participate in cell proliferation, migration and survival. This clinical case report confirms what already reported in human (31, 32) and equine (16) about the efficacy of MSCs in CMC gel on raising wound healing by promoting skin regeneration and angiogenesis. CMC is a low-cost biomaterial, already used as drug excipient, and it has power to adsorb and transport fluid, and to defend the lesion from bacterial exposure (33). Furthermore, the lack of inflammation or systemic side effects confirm the safeness of CMC and also of WJMSCs for the heterologous use. This could be ascribed to immunosuppressive properties of these cells, determined by a decreased or even absence of MHC class I-II expression, as recently observed (20).

## Conclusion

The application of WJMSCs in equine clinical practice can be encouraged by the data registered in this single case report. Indeed, no side effects associated to a fast wound regression were observed. Like in human medicine, also in veterinary medicine the constitution of cell banks would allow the application of MSCs on a large scale.

## Author Contributions

AL, JM, and CC: medical diagnosis, clinical observation, and collection of clinical data; EI: isolation, culture, and differentiation of MSCs, PCR analysis, preparation of gels and local MSCs application, interpretation of results, writing and editing the manuscript; BM: isolation, culture, and differentiation of MSCs, PCR analysis, analysis of data recorded, interpretation of results.

### Conflict of Interest Statement

The authors declare that the research was conducted in the absence of any commercial or financial relationships that could be construed as a potential conflict of interest.

## Supplementary Material

Figure S1 Foal 5 days after discharge.
