# Supplementary figures and images for: Heterologous Wharton's Jelly Derived Mesenchymal Stem Cells Application on a Large Chronic Skin Wound in a 6-Month-Old Filly
Source: Front Vet Sci. 2019 Jan 30;6:9. doi: 10.3389/fvets.2019.00009 (PMC6363668; doi:10.3389/fvets.2019.00009)

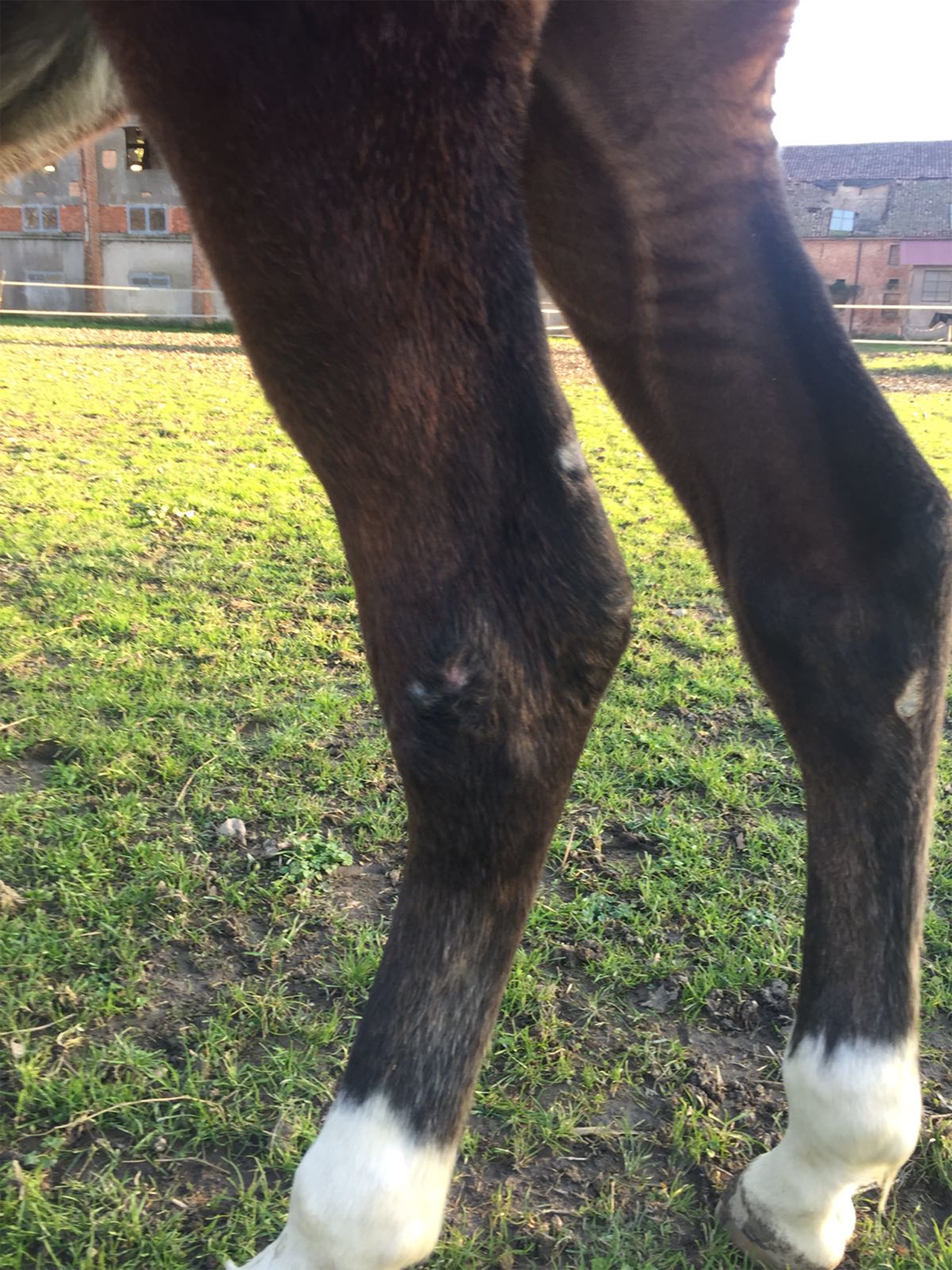

Supplement: Figure S1 — Foal 5 days after discharge. [file Image_1.TIFF]
